# Divorce conflict and health across the divorce process: A 10‐year observational study of medicine prescriptions, primary care visits and hospitalisations

**DOI:** 10.1111/bjhp.70075

**Published:** 2026-04-24

**Authors:** Andreas Nielsen Hald, Gert Martin Hald, Peter Fallesen

**Affiliations:** ^1^ Department of Public Health Aarhus University Aarhus C Denmark; ^2^ Department of Public Health University of Copenhagen København K Denmark; ^3^ Swedish Institute for Social Research Stockholm University Stockholm Sweden; ^4^ Research Unit ROCKWOOL Foundation Copenhagen C Denmark

**Keywords:** divorce conflict, divorce stress adjustment, health trajectories, healthcare use, high conflict, longitudinal study, mental health

## Abstract

**Objectives:**

This study examines the association between divorce conflict and medicine prescriptions, primary care visits and hospitalisations, over a 10‐year period around juridical divorce.

**Design:**

A longitudinal observational study was conducted using a cohort of 1784 Danes who divorced between 2015 and 2017. Conflict was measured with the validated Divorce Conflict Scale, and health outcomes were obtained from national registers.

**Methods:**

Negative binomial and logistic regression models examined the relationship between divorce conflict and health outcomes, controlling for prior health status, demographic factors and socioeconomic variables. Analyses included sensitivity tests to explore pre‐ and post‐divorce health patterns, and an exploratory analysis of health trajectories based on conflict levels.

**Results:**

A one‐standard deviation increase in divorce conflict was associated with a significant 28% increase in medicine prescriptions, a 5% increase in primary care visits, and 13% higher odds of hospitalisation in the 5 years following juridical divorce. Sensitivity analyses showed that these associations were robust but also varied depending on the pre‐divorce health period, highlighting the importance of pre‐divorce health in explaining outcomes. Exploratory analyses indicated that high‐conflict divorcees had consistently elevated health trajectories across all outcomes, with a significantly steeper increase in primary care visits before divorce compared to those with average or low conflict.

**Conclusions:**

High‐conflict divorcees experienced consistently worse health outcomes, including more medicine prescriptions, primary care visits and hospitalisations, both before and after divorce. These findings stress the importance of conceptualising divorce as a process and addressing conflict during the divorce process to mitigate long‐term health consequences.

**Statement of Contribution**

**What is already known on this subject?**

- Divorce is linked to adverse health consequences, such as mental health challenges and hospitalisation.
- Divorce conflict is consistently associated with worse health consequences of divorce.
- Current research insufficiently explores temporal patterns both before and after divorce.

**What does this study add?**

- Higher divorce conflict predicts worse health outcomes post‐divorce.
- Health issues rise pre‐divorce and stabilise post‐divorce, indicating that divorce is a process
- Divorce conflict affects health trajectories, with higher overall adverse levels for high‐conflict divorcees.

## INTRODUCTION

### Background

Divorce is a significant life transition that can affect the physical, mental and social well‐being of those involved. While experiences vary, divorcees report heightened anxiety (Hald et al., 2022), reduced subjective well‐being (Kaleta & Mróz, 2023), increased depressive symptoms (Lu et al., 2021), greater perceived stress (Strizzi et al., 2021) and higher risks of infectious diseases requiring hospitalisation (Nielsen et al., 2014). More generally, they have poorer overall physical health (Pellón‐Elexpuru et al., 2024), more socioeconomic challenges (Sbarra & Whisman, 2022) and increased mortality risk (Shor et al., 2012).

A key factor in these outcomes is the degree of conflict between partners (Hald et al., 2019). While most divorcees report some discord, 5–25 percent experience persistent high‐conflict divorces (Ciprić et al., 2022; Hald et al., 2019). High conflict involves pervasive negative interactions and a strained, hostile and distrustful emotional environment between ex‐partners. It can worsen physical, mental and social challenges (Amato, 2000, 2014; Hald et al., 2019; Ottosen et al., 2017) and is linked to higher symptoms of depression, stress and anxiety (Kalmijn & Monden, 2006; Liu & Zeng‐Yin, 2006; Symoens et al., 2014) and lower overall well‐being (Amato, 2000; Lamela et al., 2016; Symoens et al., 2014).

To better understand how divorce and divorce conflict relate to health, the Divorce‐Stress‐Adjustment Perspective (DSAP) is useful (Amato, 2000, 2014). The DSAP conceptualises divorce as a process with multiple stressors and protective factors that influence health through both selection and causation mechanisms. Stressors may already be present in the years leading up to divorce, for example shifting family dynamics or emotional distance. High conflict can intensify these stressors by creating a hostile atmosphere or can function as a stressor on its own (Booth & Amato, 2001). Low conflict may help by preserving a more supportive environment. After divorce, new demands such as co‐parenting and financial changes can extend psychological strain. High conflict may escalate these difficulties and maintain tension, whereas low conflict may support collaboration and de‐escalate these difficulties. Through a selection lens, people who experience high conflict may already differ in personality traits, psychological or physical vulnerabilities, or long‐standing strain. Through a causation lens, conflict itself may affect psychological and physiological health pathways during the divorce process.

### Present study

Existing research has conceptualised divorce as a process and documented its association with adverse health effects. Still, important knowledge gaps remain in the literature.

First, much of the existing literature relies on non‐validated measurements of divorce conflict, which limits the reliability and comparability of findings. To address this, we use the validated Divorce Conflict Scale (DCS) to provide a more precise understanding of how divorce conflict influences health outcomes and to improve the overall quality of evidence (Hald et al., 2019).

Second, most studies rely on self‐report health measures. To track yearly health consequences before and after divorce, we use objective register‐based data on medicine prescriptions, primary care visits and hospitalisations (Pellón‐Elexpuru et al., 2024; Reneflot et al., 2020). Healthcare use is widely used as a proxy for health in longitudinal research (Agerholm et al., 2016; Marselle et al., 2020; Meulman et al., 2023; Smits et al., 2009; Vedsted & Christensen, 2005). Short‐term use may reflect adaptive help‐seeking, but high or persistent use over time is associated with distress and poorer health (Smits et al., 2009; Vedsted & Christensen, 2005). To validate our outcomes, we examined their associations with depression, anxiety and somatisation from the SCL‐90‐R (Derogatis & Unger, 2010), which supported their relevance.

Third, the temporal scope of most research is limited because studies often focus only on the period after juridical divorce and overlook the earlier stages of the divorce process (Amato, 2000, 2014). To better capture this process, we use longitudinal data that cover both pre‐ and post‐divorce years, which allows us to track health trajectories across an extended time window. Ideally, we would identify exactly when the divorce process starts and ends for each couple, but this is difficult to determine, even for the individuals involved, because it may begin gradually with relational distance or emotional withdrawal. There is thus no gold standard for defining when divorce begins or ends. In this study, we therefore selected observation periods to balance data availability with the DSAP view of divorce as a process that may start years before and extend well beyond juridical divorce. Guided by this, we hypothesised that the divorce process may on average begin about 2 years before juridical divorce and extend up to 5 years after. For the main confirmatory analysis, we follow health outcomes for 5 years after juridical divorce while controlling for outcomes measured from 5 to 3 years before divorce. This provides a conservative estimate because it focuses on a period that clearly follows juridical divorce while excluding the years immediately before it, which may already reflect early stages of the process. To assess the robustness of this approach, we conducted two a priori sensitivity analyses that extend the control and outcome periods. Based on the descriptive results, we also carried out a post hoc exploratory analysis covering the entire 10‐year window using two‐ and four‐piece linear spline models.

Taken together, this study's main research question is whether higher levels of divorce conflict are linked with higher levels of healthcare use across the divorce process. In answering this, we address three important knowledge gaps by using validated conflict measures, objective register‐based outcomes, and a design that captures both pre‐ and post‐divorce periods.

## METHODS

### Study design

In this observational study, we analysed a cohort of recently divorced Danes (*n* = 1784) across a 10‐year window covering 5 years before and 5 years after each person's juridical divorce. Respondents were recruited by email through the Danish State Administration, which handled divorce decrees at the time, as part of an RCT evaluating the digital platform SES One (Ciprić et al., 2020). Survey data were collected from January 2016 to January 2018 (Hald et al., 2020). The email included a link to an online questionnaire with the DCS, which participants completed on average within 1 week of their divorce, after which they were randomly assigned to SES One or no intervention. Because respondents completed the survey after their divorce date, the exact calendar years included in each person's observation window slightly differ from the survey period. This means the divorcees in the study were divorced between 2015 and 2017, although the survey took place from January 2016 to January 2018. The Danish registers provide individual‐level data, so each participant contributed information from 5 years before to 5 years after their own divorce date. For example, a person who divorced in March 2017 contributed register data from March 2012 to March 2022.

SES One integrates elements from Cognitive Behavioural Therapy, Narrative Therapy and Acceptance and Commitment Therapy and was designed to support divorcees with post‐divorce challenges, co‐parenting and understanding children's reactions. It consists of 17 online modules (15–50 min each) that users can select freely, covering themes such as conflict management, communication with ex‐partners, children's needs and the divorcee's mental health (see Sander et al., 2024). The RCT showed that SES One reduced self‐reported depression, anxiety, stress and sick days during the first post‐divorce year (Hald et al., 2020; Sander et al., 2024).

Because randomisation in the RCT was independent of conflict level, any effects of SES One were evenly distributed across conflict groups and did not confound the association examined here. We therefore included all participants in the analyses and linked de‐identified social security numbers to national registers to obtain data on healthcare use and background variables.

### Ethical approval

As this study involved the analysis of pseudo‐anonymised data from Danish registers, it complied with the EU General Data Protection Regulation (GDPR). In accordance with the Law of Statistics Denmark, internal review board approval or participant consent was not required. The study adhered to STROBE reporting guidelines.

### Participants

The original RCT had a sample of 1856 individuals. During the 5‐year post‐divorce period, 3.1% (*n* = 58) exited the study due to migration or death. Attrition was unrelated to conflict level or background characteristics, so we treat this missingness as missing completely at random. Because outcomes were modelled as cumulative counts over defined time windows, the participants remained in the sample and contributed outcome data up until the year they migrated or died. To ensure a valid and reliable measure of divorce conflict, we excluded 68 respondents who did not answer at least one item in each conflict dimension of the DCS (61 answered none; seven answered only one) and four respondents with missing income data, resulting in a final sample of 1784 individuals. Sensitivity analyses revealed no systematic patterns among excluded respondents.

All respondents were Danish and divorced in Denmark, which has one of the most liberal divorce laws in the world (Rosenbeck, 2017). Couples can typically separate without court involvement, and juridical divorce is granted administratively through the Agency of Family Law (prior to 2019, this was handled by the Danish State Administration). In cases involving children, parents are encouraged or required to participate in mediation before finalising co‐parenting plans. This legal framework makes it relatively easy to exit a marriage. Still, prior research has shown that even in countries like Denmark, divorce stressors can lead to prolonged interparental disputes and negatively affect post‐divorce well‐being (Hald et al., 2019; Ottosen et al., 2017).

Table 1 shows most respondents were female (67.3%), had education above high school (62.5%) and were born to at least one parent with Danish citizenship (91.8%). The average age was 45.1 years, and average income was DKK 479,372. Marriages lasted an average of 12.7 years, with 84% of divorces initiated by one spouse alone and 36% of respondents having a new partner at the time of divorce.

**TABLE 1:** Descriptive statistics of the sample on background, predictor and outcome variables.

Background variables	Total *N* (%)
Total	1784 (100)
Initiated divorce	
Respondent	784 (44.0)
Both	280 (15.7)
Former spouse	720 (40.4)
New partner	
Both	87 (4.8)
None	1136 (63.7)
Respondent	185 (10.4)
Former spouse	376 (21.1)
Number of children	
0	169 (9.5)
1	287 (16.1)
2	920 (51.0)
3	253 (19.8)
4	60 (3.4)
5	4 (.2)
6	1 (.1)
Times divorced	
1	1572 (88.1)
2	178 (10.0)
3	27 (1.5)
4	7 (.4)
Danish origin^a^	
No	146 (8.2)
Yes	1638 (91.8)
Educational attainment	
Short (ISCED 0–2)	125 (7.0)
Medium (ISCED 3–4)	544 (30.5)
Long (ISCED 5+)	1115 (62.5)
Divorce month	
Jan	169 (9.5)
Feb	150 (8.4)
Mar	175 (9.8)
Apr	133 (7.5)
May	141 (7.9)
Jun	132 (7.4)
Jul	162 (9.1)
Aug	161 (9.0)
Sep	138 (7.7)
Oct	155 (8.7)
Nov	146 (8.2)
Dec	122 (6.8)
Divorce year	
2015	87 (4.9)
2016	917 (51.4)
2017	780 (43.7)
Age^b^	45.104 (8.541)
Child age^b^	13.514 (8.176)
Duration of marriage^b^	12.727 (7.983)
Income (in DKK)^b^	479,372 (325,301)

Predictor variable (min, max)	Total Mean (std.dev)
Conflict level	
DCS (6, 27)	13.794 (4.880)
Latent IRT variable (−2.232, 2.622)	.000 (1.000)

Health outcomes	Total Mean (std.dev)
Medicine prescriptions	
Counting from 0 to +5 relative to divorce	4.881 (16.869)
Counting from −2 to +5 relative to divorce	6.302 (21.920)
Counting from −5 to −3 relative to divorce	1.254 (5.057)
Counting from −5 to −2 relative to divorce	1.929 (7.617)
Primary care visits	
Counting from 0 to +5 relative to divorce	62.854 (53.879)
Counting from −2 to +5 relative to divorce	87.725 (70.646)
Counting from −5 to −3 relative to divorce	30.308 (28.849)
Counting from −5 to −2 relative to divorce	42.465 (37.335)
Ever hospitalised (0 = no, 1 = yes)	
Counting from 0 to +5 relative to divorce	.256 (.436)
Counting from −2 to +5 relative to divorce	.322 (.467)
Counting from −5 to −3 relative to divorce	.128 (.334)
Counting from −5 to −2 relative to divorce	.183 (.387)

Abbreviation: ISCED: International Standard Classification of Education.

^a^ Danish origin coded as at least one parent of the respondent had Danish citizenship.

^**b**^ Age, Child age, Duration of marriage, and Income are continuous variables presented as mean (std.dev.).

### Procedures and Measures

The institutions managing the registers compiled and organised the data. We submitted requests for specific data and merged the datasets using social security identifiers. Data requests, organisation and administration were conducted from December 2023 to August 2024, with the final dataset completed by September 2024.

Data on respondents' social security numbers, treatment status, and marriage‐related variables were sourced from the RCT study. Background variables, including legal gender (man/woman), age (in years), income (total pre‐tax income in DKK), educational level (highest completed), migration background (Danish or non‐Danish origin) and date of juridical divorce, were obtained from Statistics Denmark's Population Register, Income Register and Labor Market Register, measured at juridical divorce.

Information on divorce conflict was sourced from responses to the DCS in the RCT study (Ciprić et al., 2020; Hald et al., 2019). The DCS identifies three interconnected dimensions to conceptualise divorce conflict (Anderson et al., 2010; Hald et al., 2019; Johnston, 1994; Ottosen et al., 2017). The domain dimension refers to the areas where disagreements occur, such as child‐rearing, custody arrangements, financial matters and emotional issues. The tactics dimension reflects the methods and strategies used to address or solve these disagreements, ranging from collaborative approaches to hostile strategies like threats or force. The attitudinal dimension captures the degree of negativity, distrust and hostility between former spouses. The DCS consists of six items, please see Hald et al. (2019) for full details on the items and response categories.

An Item Response Theory Graded Response Model (IRT‐GRM) was used to generate a standardised latent variable for divorce conflict, used as the predictor in all analyses. This approach accounted for differing scales, accommodated the ordinal nature of the six Likert‐scale DCS items, and handled missing responses under the assumption that they were missing at random, consistent with the treatment of ignorable missing data in IRT models (Sulis & Porcu, 2017). Missing data primarily affected one DCS item: “My former spouse and I have no trouble talking about issues concerning our child/children,” which was not administered to 49% of the sample due to technical issues in the RCT. All items showed acceptable discrimination (1.74–2.83) and threshold parameters, with high reliability (marginal reliability = .84). Latent scores (theta) ranged from −2.23 to +2.62, with higher scores indicating greater conflict. Unidimensionality was confirmed via confirmatory factor analysis, with good fit (RMSEA = .047, CFI = .992, TLI = .987, CD = .890) and factor loadings (.454–.796).

Outcome variables were sourced from the Danish Patient Register, Medical Insurance Database and Medical Prescription Database. ‘Medicine prescriptions’ included all filled prescriptions based on ATC codes N05 (psycholeptics, including antipsychotics, anxiolytics, hypnotics and sedatives) and N06A (antidepressants). N06C (psycholeptics and psychoanaleptics in combination) was excluded, as it is not used in Denmark. ‘Primary care visits’ included all billed consultations with publicly funded general practitioners, specialist practitioners and psychologists. ‘Hospitalisations’ were measured as a binary variable indicating whether an individual spent at least one night in the hospital.

To assess the validity of the health outcomes as proxies for health status, we examined their associations with standardised scores on the depression, anxiety, and somatisation subscales of the SCL‐90‐R, measured at the time of juridical divorce. As shown in Table S1, a one‐standard‐deviation increase in depression was associated with 42.4% more medicine prescriptions and 15.7% more primary care visits; anxiety with 57.2% more prescriptions and 18.5% more visits; and somatisation with 47.9% more prescriptions, 22.1% more visits and 25.5% higher odds of hospitalisation. These results indicate that the outcomes are strongly linked to validated measures of self‐perceived health, and we therefore interpret them as valid proxies for health status.

### Statistical methods

Analyses were conducted using Stata/MP 18.0 (StataCorp 2023). Two‐tailed tests were used, with α = .05 considered statistically significant.

To examine associations between divorce conflict and the count outcomes (medicine prescriptions and primary care visits), we used separate negative binomial regression models. Additional models were run for the individual variables of each index (psycholeptics, antidepressants; general practitioners, specialists, psychologists) to assess differential associations. All models had log‐dispersion parameters significantly above zero, confirming overdispersion and the appropriateness of negative binomial regression. Results are presented as Incidence Rate Ratios (IRRs) with corresponding confidence intervals and *p*‐values.

To examine the association between divorce conflict and hospitalisations, we used logistic regression. This method was chosen over negative binomial regression due to the high number of zeros in the data, with 77% of respondents not hospitalised during the 5 years after juridical divorce. Results are presented as odds ratios with corresponding confidence intervals and *p*‐values.

Because divorce is a process and the onset of divorce‐related stressors varies across individuals, it is uncertain when changes in health begin, meaning that estimated associations may differ depending on how the pre‐ and post‐divorce windows are defined. To address this uncertainty and control for confounding by prior health status, we conducted a confirmatory a priori main analysis addressing the primary research question, along with two a priori sensitivity analyses to test robustness. After reviewing descriptive plots of observed yearly means, we also carried out post hoc exploratory analyses of health trajectories.

Relative to juridical divorce, the main analysis counted outcomes from year 0 to +5, controlling for years −5 to −3; Sensitivity Analysis 1 used the same outcome period but controlled for years −5 to −2; and Sensitivity Analysis 2 counted outcomes from year −2 to +5, controlling for years −5 to −3. If the results change across these analyses, it will indicate that timing uncertainty affects how the findings should be interpreted. It would also suggest that the conclusions depend heavily on how we choose to define the ‘divorce window’.

To further examine the association between divorce conflict and health outcomes, we predicted marginal effects of the continuous conflict variable at .5‐SD increments. Predicted values were obtained using average marginal predictions from Stata's margins command, which fixes the conflict variable at specified values while averaging predicted outcomes over the observed distribution of all other covariates. To improve interpretability for medicine prescriptions, control variables for prior medicine use were log‐transformed (with +1 added to handle zeros). This stabilised the models and resolved issues with inflated margins due to outliers. The transformation did not affect predictor coefficients, p‐values or the overall interpretation.

We also explored health trajectories over the 10‐year period by plotting observed mean values for three conflict groups: low (<−1 SD), average (−1 to +1 SD) and high (>+1 SD) on the standardised IRT conflict variable. Conflict was analysed as a continuous variable in all other models; the low, average and high‐conflict groups are used only to aid interpretation in the exploratory analysis. Given the standardisation, about 16% of the sample fell into each of the low‐ and high‐conflict groups, and 68% into the average group, consistent with prior research on high‐conflict prevalence (Ciprić et al., 2022; Hald et al., 2019). To further examine potential linear or curvilinear trends, we estimated two‐piece linear spline models (pre/post‐divorce) and four‐piece models with breakpoints at −2 and + 2 years (Wright & London, 2009). Predicted values were plotted alongside observed means. Spline models used negative binomial regression for prescriptions and primary care visits, and logistic regression for hospitalisations.

Finally, in all models we tested an interaction between intervention assignment and divorce conflict. It was not significant in any model and did not improve model fit, so it was not included.

## RESULTS

Conflict was analysed as a continuous variable in the main and sensitivity analyses. We present average marginal predictions from the fully adjusted model, estimated at selected values of the continuous conflict variable and averaged over the observed distribution of all other covariates. For the main interpretation of our results, we focus on the average marginal predictions at +/− 1 SD from the mean of the conflict variable. In the exploratory analyses, conflict was instead analysed as a categorical variable. The categories “low conflict” (<−1 SD), “average conflict” (−1 to +1 SD) and “high conflict” (>+1 SD) are derived from the continuous conflict variable.

### Main analysis: Health outcomes from divorce to 5 years after, controlling for outcomes from 5 to 3 years before

**FIGURE 1:**
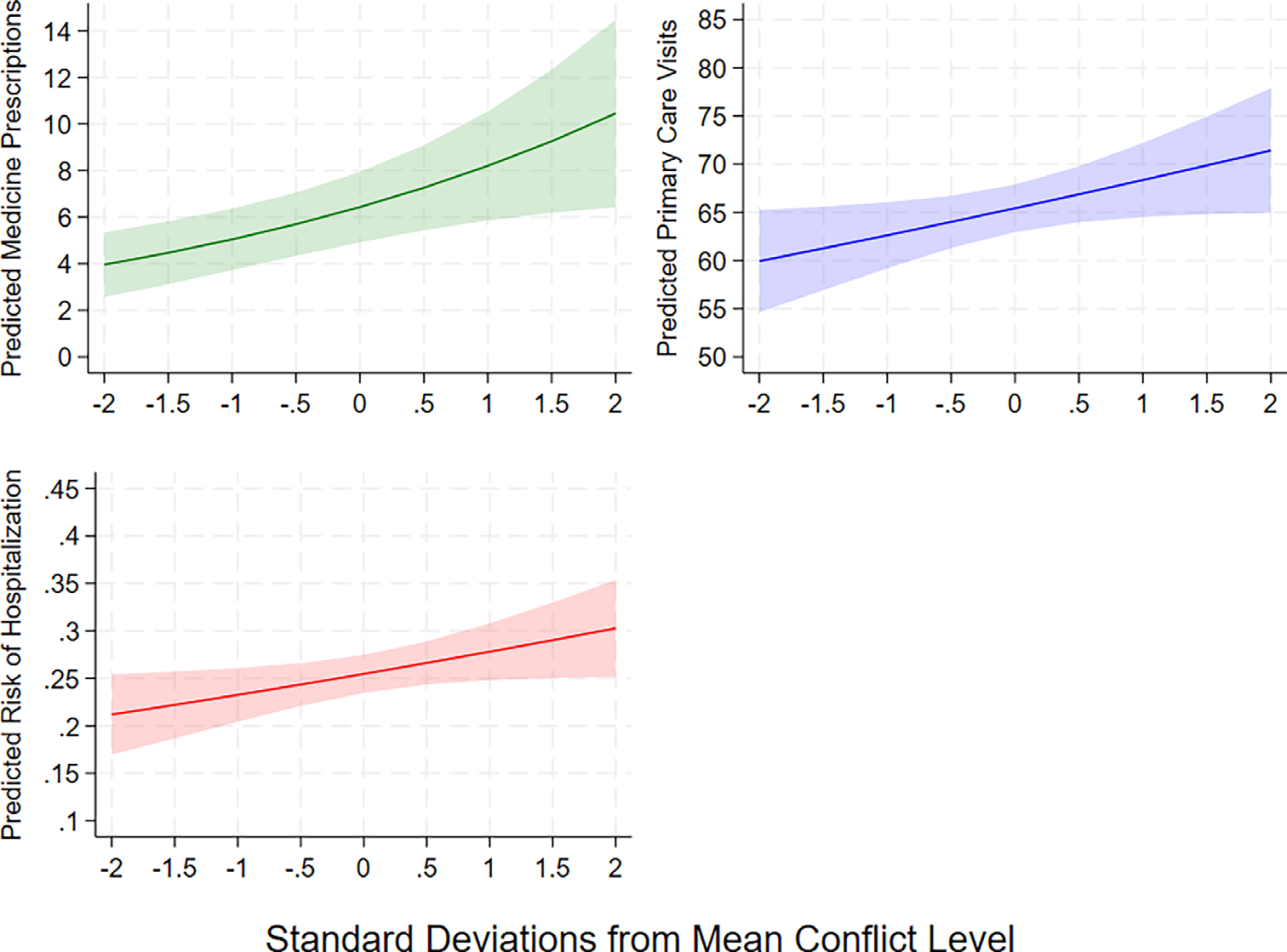
Average Marginal Predicted Medicine Prescriptions, Primary Care Visits, and Hospitalisations by Conflict Level, with 95% Confidence Intervals (*N* = 1784). Average Marginal Predicted Values for the Individual Variables of Each Index are Provided in Figure S1. Predicted Margins Were Calculated for the Continuous Conflict Variable at .5‐SD Increments from −2 to 2, with Confidence Intervals Reflecting These Discrete Prediction Points.

Higher divorce conflict was significantly associated with increased medicine prescriptions in the 5 years post‐divorce (Table 2: IRR = 1.275, 95% CI: 1.106–1.469, *p* = .001), with a one‐standard‐deviation increase in conflict corresponding to a 27.5% higher expected count of prescriptions. Respondents at +1 SD had about three more average marginal predicted prescriptions than those at −1 SD (−1 SD = 5.05; +1 SD = 8.21, Figure 1). This effect was primarily driven by psycholeptics (Table S3: IRR = 1.572, 95% CI: 1.308–1.889, *p* < .001), with a non‐significant association for antidepressants.

**TABLE 2:** Divorce conflict's association with medicine prescriptions, primary care visits, and hospitalisations from divorce to 5 years after, controlling for outcomes from 5 to 3 years before divorce (*N* = 1784).

Health outcome	IRR/OR	95% CI	*p*
Medicine prescriptions (years 0 to 5)	*IRR*		
Conflict level	1.275	1.106–1.469	.001
Medicine prescriptions (years −5 to −3)	3.225	2.824–3.683	<.001
Primary care visits (years 0 to 5)	*IRR*		
Conflict level	1.045	1.003–1.088	.034
Primary care visits (years −5 to −3)	1.014	1.012–1.015	<.001
Ever hospitalised (years 0 to 5)	*OR*		
Conflict level	1.131	1.009–1.269	.035
Ever hospitalised (years −5 to −3)	2.436	1.813–3.273	<.001

*Note*: All presented coefficients are estimated from the full models adjusted for divorce initiator, age, gender, income and education. All coefficients from the full models are provided in Table S2 and models for the individual variables of each index are provided in Table S3. Medicine prescriptions and primary care visits were analysed using negative binomial regression, with exponentiated coefficients reported as incidence rate ratios (IRRs). Hospitalisations (binary outcome) were analysed using logistic regression, with exponentiated coefficients reported as odds ratios (ORs). Medicine prescriptions (years −5 to −3) were log‐transformed.

Divorce conflict was also positively associated with primary care visits in the 5 years post‐divorce (Table 2: IRR = 1.045, 95% CI: 1.003–1.088, *p* = .034), with a one‐standard‐deviation increase in conflict corresponding to a 4.5% increase in expected visits. Respondents at +1 SD had approximately six more average marginal predicted visits than those at −1 SD (−1 SD = 62.63; +1 SD = 68.36, Figure 1). This was mainly driven by psychologist visits (Table S3: IRR = 1.237, 95% CI: 1.068–1.434, *p* = .005), with non‐significant associations for GP and specialist visits.

Finally, higher conflict was associated with increased odds of hospitalisation (Table 2: OR = 1.131, 95% CI: 1.009–1.269, *p* = .035), with a one‐standard deviation increase in divorce conflict corresponding to a 13.1% increase in hospitalisation odds. Respondents at +1 SD had a 5‐percentage‐point higher average marginal predicted probability of hospitalisation than those at −1 SD in the 5 years post‐divorce (−1 SD = .23; +1 SD = .28, Figure 1).

### Sensitivity analysis 1: Health outcomes from divorce to 5 years after, controlling for outcomes from 5 to 2 years before

When controlling for outcomes up to 2 years before juridical divorce, higher divorce conflict remained significantly associated with increased medicine prescriptions (Table 3: IRR = 1.193, 95% CI: 1.037–1.373, *p* = .014) and hospitalisation (OR = 1.126, 95% CI: 1.003–1.265, *p* = .045) in the 5 years post‐divorce. However, these associations were weaker than in the main analysis, with IRR and OR values reduced by .082 and .005, respectively. More notably, the association with primary care visits (IRR = 1.035, 95% CI: .996–1.075, *p* = .075) was just short of the predefined significance threshold (*p* < .05).

**TABLE 3:** Divorce conflict's association with medicine prescriptions, primary care visits and hospitalisations from divorce to 5 years after, controlling for outcomes from 5 to 2 years before divorce (N = 1784).

Health outcome	IRR/OR	95% CI	*p*
Medicine prescriptions (years 0–5)	*IRR*		
Conflict level	1.193	1.037–1.373	.014
Medicine prescriptions (years −5 to −2)	3.008	2.704–3.347	<.001
Primary care visits (years 0–5)	*IRR*		
Conflict level	1.035	.996–1.075	.075
Primary care visits (years −5 to −2)	1.012	1.011–1.013	<.001
Ever hospitalised (years 0–5)	*OR*		
Conflict level	1.126	1.003–1.265	.045
Ever hospitalised (years −5 to −2)	2.681	2.066–3.478	<.001

*Note*: All presented coefficients are estimated from the full models adjusted for divorce initiator, age, gender, income and education. All coefficients from the full models are provided in Table S4 and models for the individual variables of each index are provided in Table S5. Medicine prescriptions and primary care visits were analysed using negative binomial regression, with exponentiated coefficients reported as incidence rate ratios (IRRs). Hospitalisations (binary outcome) were analysed using logistic regression, with exponentiated coefficients reported as odds ratios (ORs). Medicine prescriptions (years −5 to −2) were log‐transformed.

### Sensitivity analysis 2: Health outcomes from 2 years before divorce to 5 years after, controlling for outcomes from 5 to 3 years before

When counting health outcomes from 2 years before to 5 years after juridical divorce, higher divorce conflict was significantly associated with increased prescriptions (Table 4: IRR = 1.271, 95% CI: 1.105–1.462, *p* = .001), with an IRR nearly identical to the main analysis (.004 lower). The association with primary care visits was also significant (IRR = 1.039, 95% CI: 1.003–1.076, *p* = .033), differing only slightly from the main analysis (.006 lower IRR). Conflict was also significantly associated with hospitalisation (OR = 1.173, 95% CI: 1.055–1.304, *p* = .003), with a notably stronger effect than in the main analysis (.042 higher OR).

**TABLE 4:** Divorce conflict's association with medicine prescriptions, primary care visits, and hospitalisations from 2 years before divorce to 5 years after, controlling for outcomes from 5 to 3 years before divorce (*N* = 1784).

Health outcome	IRR/OR	95% CI	*p*
Medicine prescriptions (years −2 to 5)	*IRR*		
Conflict level	1.271	1.105–1.462	.001
Medicine prescriptions (years −5 to −3)	3.503	3.086–3.976	<.001
Primary care visits (years −2 to 5)	*IRR*		
Conflict level	1.039	1.003–1.076	.033
Primary care visits (years −5 to −3)	1.014	1.013–1.016	<.001
Ever hospitalised (years −2 to 5)	*OR*		
Conflict level	1.173	1.055–1.304	.003
Ever hospitalised (years −5 to −3)	2.239	1.672–2.997	<.001

*Note*: All presented coefficients are estimated from the full models adjusted for divorce initiator, age, gender, income and education. All coefficients from the full models are provided in Table S6 and models for the individual variables of each index are provided in Table S7. Medicine prescriptions and primary care visits were analysed using negative binomial regression, with exponentiated coefficients reported as incidence rate ratios (IRRs). Hospitalisations (binary outcome) were analysed using logistic regression, with exponentiated coefficients reported as odds ratios (ORs). Medicine prescriptions (years −5 to −3) were log‐transformed.

### Exploratory analysis: Health trajectories by divorce conflict level

**FIGURE 2:**
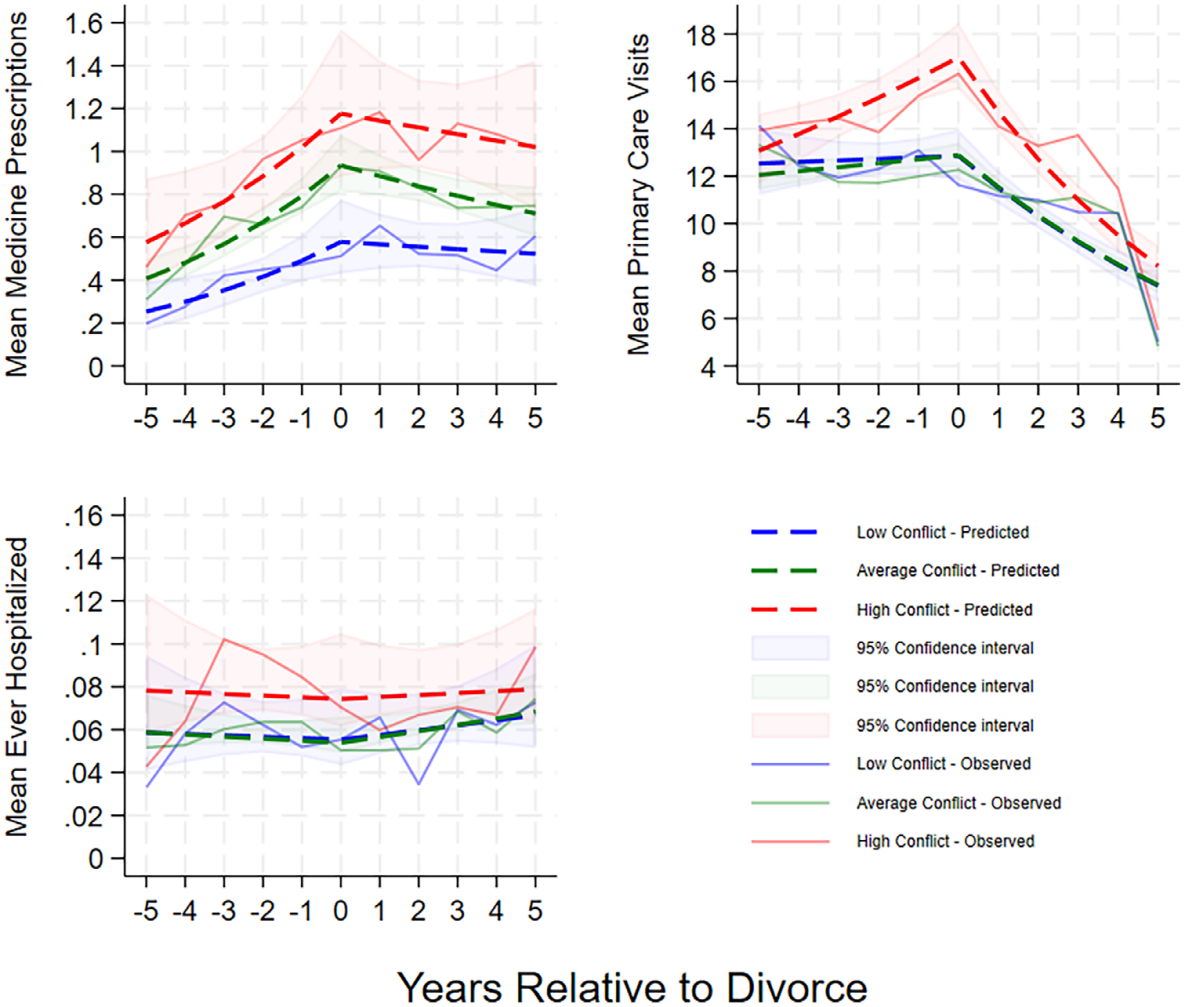
Yearly Predicted and Observed Means of Medicine Prescriptions, Primary Care Visits, and (Binary) Hospitalisations by Conflict Group (*N* = 1784). Yearly Predicted and Observed Means of the Individual Variables of Each Index are Provided in Figure S2. Predicted Values Are Based on Two‐Piece Linear Spline Models with Separate Slopes Before and After Juridical Divorce (Year 0).

Medicine prescriptions increased significantly before divorce in the low‐conflict group (Table 5: IRR = 1.195, 95% CI: 1.059–1.349, *p* = .004) and levelled off afterward (Table 5: IRR = .980, 95% CI: .886–1.083, *p* = .687). High conflict was associated with substantially higher overall prescription levels (Table 5: IRR = 1.953, 95% CI: 1.299–2.936, *p* = .001), but the rate of change before and after divorce did not differ by conflict level, suggesting parallel health trajectories at different levels, as shown in Figure 2. Figure S2 shows larger pre‐divorce differences in antidepressants and larger post‐divorce differences in psycholeptics between conflict levels, though these were not reflected in differing rates of change (Table S8).

**TABLE 5:** Divorce conflict's association with medicine prescriptions, primary care visits and hospitalisations, modelled using interactions between conflict level and two‐piece linear splines for time before and after divorce (*N* = 1784).

	Medicine prescriptions IRR (CI 95%), *p*‐value	Primary care visits IRR (CI 95%), *p*‐value	Hospitalisations OR (CI 95%), *p*‐value
Conflict level main effect			
Average conflict vs. low conflict	**1.560 (1.130 to 2.155), *p* = .007**	.988 (.894 to 1.067), *p* = .605	.969 (.692 to 1.358), *p* = .856
High conflict vs. low conflict	**1.953 (1.299 to 2.936), *p* = .001**	**1.250 (1.116 to 1.399), *p* < .001**	1.317 (.878 to 1.977), *p* = .183
Pre‐divorce slope for low conflict	**1.195 (1.059 to 1.349), p = .004**	1.007 (.976 to 1.039), *p* = .662	.988 (.876 to 1.113), *p* = .837
Post‐divorce slope for low conflict	.980 (.886 to 1.083), p = .687	.**892 (.867 to .918), p < .001**	1.041 (.939 to 1.154), *p* = .445
Conflict level × pre‐divorce slope			
Average conflict vs. low conflict	.992 (.868 to 1.133), *p* = .900	1.008 (.973 to 1.043), *p* = .671	.994 (.870 to 1.135), *p* = .924
High conflict vs. low conflict	.941 (.796 to 1.112), *p* = .475	**1.048 (1.003 to 1.095), *p* = .037**	1.002 (.854 to 1.176), *p* = .979
Conflict level × post‐divorce slope			
Average conflict vs. low conflict	.973 (.871 to 1.088), *p* = .636	.998 (.967 to 1.031), *p* = .904	1.011 (.901 to 1.133), *p* = .853
High conflict vs. low conflict	.989 (.859 to 1.138), *p* = .878	.966 (.928 to 1.007), *p* = .101	.973 (.847 to 1.118), *p* = .700

*Note*: *p* < .05 highlighted in bold. “×” denotes an interaction term that reflects whether the slope for each time segment differs by conflict level compared to the low‐conflict group. All presented coefficients are estimates from the full models adjusted for divorce initiator, age, gender, income and education. Models for the individual variables of each index are provided in Table S8. Medicine prescriptions and primary care visits were analysed using negative binomial regression with exponentiated coefficients reported as Incidence Rate Ratios (IRRs); hospitalisations (binary outcome) were analysed using logistic regression with exponentiated coefficients reported as Odds Ratios (ORs). All models include interactions between conflict level (low, average, high) and two‐piece linear spline time variables (pre‐divorce and post‐divorce slopes).

For primary care visits, levels were stable before divorce in the low‐conflict group, followed by a significant decline post‐divorce (Table 5: IRR = .892, 95% CI: .867–.918, *p* < .001), likely reflecting reduced use during the COVID‐19 pandemic in years 4 and 5. The high‐conflict group had a higher overall level of visits (Table 5: IRR = 1.250, 95% CI: 1.116–1.399, *p* < .001) and a steeper pre‐divorce increase (Table 5: IRR = 1.048, 95% CI: 1.003–1.095, *p* = .037), suggesting an escalation in visits leading up to divorce, as evidenced by Figure 2. Figure S2 shows this pattern was consistent across all variables. Table S8 indicates some pre‐divorce slope differences, especially for psychologist visits, but none were statistically significant.

For hospitalisation, patterns were erratic, and no significant associations with conflict level were found in any model term. Pre‐ and post‐divorce slopes were non‐significant across all groups, and differences in overall levels did not reach significance. This suggests that, unlike prescriptions and primary care visits, hospitalisation odds were not meaningfully influenced by divorce conflict in the two‐piece spline model.

Lastly, we tested four‐piece linear spline models with breakpoints at −2 and + 2 years relative to juridical divorce to explore finer patterns in health trajectories. Results (Figures S3 and S4) were similar to the two‐piece models but indicated a potential breakpoint around 2 years pre‐divorce, particularly for primary care visits. However, increased multicollinearity between slope terms led to less stable estimates, and the added complexity did not appear to improve explanatory value. We therefore do not consider the four‐piece models an improvement over the two‐piece models.

## DISCUSSION

### Principal findings

This study shows a clear and persistent association between divorce conflict and divorcees' health. Higher conflict was linked to worse outcomes across all measures. In line with the DSAP (see Introduction), the results support the idea of ‘divorce as a process’ that often starts years before the actual break‐up itself rather than ‘divorce as a discrete event’ such as the juridical divorce.

A one‐standard‐deviation increase in divorce conflict was linked to 28% more medicine prescriptions, 5% more primary care visits, and 13% higher odds of hospitalisation in the 5 years after juridical divorce. Sensitivity analysis 1 shows that part of the association may be explained by the period from 3 to 2 years before divorce. Sensitivity analysis 2 shows that associations with medicine prescriptions and primary care visits were similar before and after divorce, while the association with hospitalisations was stronger pre‐divorce. Exploratory analyses suggest that high‐conflict divorcees had consistently elevated health trajectories, with a steeper rise in primary care visits before divorce and otherwise similar trajectories as low or average conflict.

### Patterns and mechanisms linking divorce conflict and health outcomes

The patterns of medicine prescriptions showed that across all conflict groups, prescriptions increased leading up to the juridical divorce and then stabilised. However, low‐conflict divorcees consistently had fewer prescriptions and high‐conflict divorcees consistently had more. The pre‐divorce differences in medicine prescriptions among low‐, average‐ and high‐conflict groups appeared to be driven by higher pre‐divorce use of antidepressants, whereas post‐divorce differences appeared more driven by psycholeptics. Depressive symptoms have been linked to escalating conflict before divorce (Hald et al., 2019; Kalmijn & Monden, 2006), explaining the pre‐divorce pattern in antidepressants. In contrast, the stress persisting after high‐conflict divorces (Cummings & Davies, 1996; Reneflot et al., 2020) may lead to sleep difficulties and anxiety, explaining the pattern in psycholeptics. This interpretation is supported by the main analysis and both sensitivity analyses, where conflict level was significantly associated with increased psycholeptics use in the 5 years after divorce but not antidepressants. This pattern suggests that antidepressants may reflect a selection process, identifying individuals already at risk of high‐conflict divorces, whereas psycholeptics align more with the causation perspective (Amato, 2000).

The patterns of primary care visits showed that although visit rates were relatively stable in the years leading up to divorce, they rose sharply from 2 years before the juridical divorce through the divorce year itself, particularly among individuals in the high‐conflict group. Understanding divorce as a process that begins before juridical divorce, this rise may reflect a causation perspective. High conflict may exacerbate psychological distress and prompt increased help‐seeking during a period of acute stress and adjustment (Lamela et al., 2016; Symoens et al., 2014). In the main analysis, divorce conflict was significantly associated with increased post‐divorce visits, particularly to psychologists; this association held in sensitivity analysis 2 but not in sensitivity analysis 1. This suggests that the timing and level of conflict contribute to increased primary care visits, consistent with a causation perspective. The gradual decline in visit rates across all conflict groups post‐divorce also suggests some stabilisation over time, with convergence by the end of the five‐year period. However, this was likely accelerated by the COVID‐19 pandemic, during which non‐acute GP consultations were largely suspended and many Danes reduced or delayed healthcare‐seeking due to lockdowns and infection concerns (Olagnier & Mogensen, 2020; Raasthøj Holst et al., 2025).

The patterns of hospitalisation were relatively stable across the 10‐year period, with no significant upward or downward predicted slopes leading up to or following juridical divorce across conflict groups. Nevertheless, the odds of hospitalisation increased with conflict level, with the high‐conflict group showing the highest odds. Differences were most pronounced in the pre‐divorce years, whereas post‐divorce patterns were less clear. In the main analysis, divorce conflict was significantly associated with increased odds of hospitalisation. This association remained significant in both sensitivity analyses, although it was substantially stronger in sensitivity analysis 2. This suggests that hospitalisations may reflect a selection process, where individuals who were hospitalised before divorce are also more likely to go through high‐conflict divorce. At the same time, the lack of a consistent post‐divorce increase makes a strong causation explanation less likely.

### Practical implications

The observed rise in primary care visits and prescriptions in the years before juridical divorce, especially among high‐conflict divorcees, suggests that the primary healthcare workforce could screen patients who present with stress‐related symptoms for relationship strain or conflict. Simple brief screeners on relationship quality may help identify those experiencing high conflict and enable earlier referral to mediation, co‐parenting counselling and digital health platforms (Becher et al., 2015; O'Hara et al., 2024; Turner et al., 2021). More broadly, because high‐conflict divorcees showed persistently elevated health trajectories across the full 10‐year period, initiatives to improve interprofessional collaboration within primary care (Hald et al., 2021) and coordination across sectors such as primary care and social care (Kristensen et al., 2019) would also be essential to secure continuity of support for these at‐risk individuals. Importantly, whether it is a new screening tool or way to collaborate, it is essential to involve the stakeholders who are expected to implement these initiatives, as their involvement helps surface tensions that can hinder uptake (Hald et al., 2025) and makes it easier to identify the key conditions for success (Hald et al., 2024).

### Limitations and future research

First, while medicine prescriptions, primary care visits, and hospitalisations provide objective, longitudinal health indicators, they may also partly reflect short‐term help‐seeking that supports recovery. Future research may consider more direct measures such as repeated self‐ratings or biomarkers, or longer‐term indicators such as sick leave, disability benefits or mortality.

Second, register data strengthens reliability but does not capture the lived experience of divorce conflict. Qualitative research such as interview studies could help clarify how people understand and manage conflict and how this shapes health status, offering insight into coping strategies and the social and emotional consequences of conflict.

Third, we could not definitively determine where each couple was in the divorce process. We addressed this with sensitivity and exploratory analyses across multiple periods, but any defined period remains a proxy.

Fourth, we used the DCS as a complete scale to measure divorce conflict. It is possible that the domain, tactics and attitudinal components relate differently to health outcomes. Future research could examine these dimensions separately or compare results using alternative divorce conflict measures to assess robustness.

Fifth, we adjusted for prior healthcare use, demographics, socioeconomic status and divorce initiation, but residual confounding likely remains. Unmeasured factors such as personality or social factors like the presence and age of children, family estrangement or parental alienation may influence both conflict and health. These factors are closely intertwined with the divorce process and were therefore not included as control variables, but they remain important contextual elements that future studies designed to separate them more clearly from divorce conflict could explore. We also lacked data on the former spouse's gender, although same‐sex marriages made up only 1.7% of our sample, limiting meaningful subgroup analysis.

Sixth, the sample was drawn from a Danish population, which may limit generalisability. Denmark's universal healthcare, liberal divorce laws and general acceptance of divorce may buffer the health impact of conflict (Birk et al., 2024). In settings with less healthcare access or different cultural norms, the effects may differ, as stigma and social expectations affect both the experience of conflict and its health consequences. Cross‐cultural studies could help clarify how legal, social and cultural factors influence this association.

Finally, the COVID‐19 pandemic likely affected healthcare use in the later years of our observation window. Denmark's early lockdowns and rapid reorganisation of healthcare services changed access to primary care and led many Danes to delay or avoid seeking care (Olagnier & Mogensen, 2020; Raasthøj Holst et al., 2025). Divorce filings also declined to their lowest level in 5 years (Fallesen, 2021). These factors suggest that the decline in primary care visits in years four and five post‐divorce likely reflects pandemic‐related disruptions.

## AUTHOR CONTRIBUTIONS

**Andreas Nielsen Hald:** Writing – original draft; writing – review and editing; formal analysis; methodology; conceptualization. **Gert Martin Hald:** Conceptualization; funding acquisition; supervision; writing – review and editing. **Peter Fallesen:** Data curation; formal analysis; methodology; funding acquisition; writing – review and editing.

## FUNDING INFORMATION

Funding for the study was provided by the ROCKWOOL Foundation (grant no. 1263). The RCT study from which the sample cohort was generated was funded by the Egmont Foundation and the Carlsberg Foundation (grant no. CF16‐0094, Gert Martin Hald).

## CONFLICT OF INTEREST STATEMENT

For due diligence, we would like to declare that the co‐author GMH is a partner and co‐owner of a company currently holding the commercial licence and intellectual property rights to a digital platform for parents and children experiencing parental relationship dissolution. GMH has not decided or had final say on data analyses or the results reported. ANH and PF declare no competing interests.

## Supporting information


**Table S1.** Associations Between Self‐Rated Somatization, Depression, and Anxiety (SCL‐90‐R Subscales) and Medicine Prescriptions, Primary Care Visits, and Hospitalisations at the Year of Juridical Divorce (*N* = 1740).
**Table S2**. Divorce conflict’s association with medicine prescriptions, primary care visits, and hospitalisations from divorce to 5 years after, controlling for outcomes from 5 to 3 years before divorce (*N* = 1784).
**Table S3**. Divorce Conflict’s Association with Psycholeptics, Antidepressants, GP Visits, Specialist Visits, and Psychologist Visits from Divorce to 5 Years After, controlling for Outcomes from 5 to 3 Years Before Divorce (*N* = 1784).
**Table S4**. Divorce conflict’s association with medicine prescriptions, primary care visits, and hospitalisations from divorce to 5 years after, controlling for outcomes from 5 to 2 years before divorce (*N* = 1784).
**Table S5**. Divorce Conflict’s Association with Psycholeptics, Antidepressants, GP Visits, Specialist Visits, and Psychologist Visits from Divorce to 5 Years After, controlling for Outcomes from 5 to 2 Years Before Divorce (*N* = 1784).
**Table S6**. Divorce conflict’s association with medicine prescriptions, primary care visits, and hospitalisations from 2 years before divorce to 5 years after, controlling for outcomes from 5 to 3 years before divorce (*N* = 1784).
**Table S7**. Divorce Conflict’s Association with Psycholeptics, Antidepressants, GP Visits, Specialist Visits, and Psychologist Visits from 2 Years Before Divorce to 5 Years After, controlling for Outcomes from 5 to 3 Years Before Divorce (*N* = 1784).
**Table S8**. Divorce Conflict’s Association with Psycholeptics, Antidepressants, GP Visits, Specialist Visits, and Psychologist Visits, Modelled Using Interactions Between Conflict Level and Two‐Piece Linear Splines for Time Before and After Divorce (*N* = 1784).
**Figure S1**. Average Marginal Predicted Psycholeptics Prescriptions, Antidepressants Prescriptions, GP Visits, Specialist Visits, and Psychologist Visits by Conflict Level, with 95% Confidence Intervals (*N* = 1784).
**Figure S2**. Yearly Predicted and Observed Means of Psycholeptics, Antidepressants, GPs, Specialists, and Psychologist Visits by Conflict Group (N = 1784). Predicted Values Are Based on Two‐Piece Linear Models With Separate Slopes Before and After Juridical Divorce (Year 0).
**Figure S3**. Yearly Predicted and Observed Means of Medicine Prescriptions, Primary Care Visits, and Binary Hospitalisations by Conflict Group (*N* = 1784). Predicted Values are Based on Four‐Piece Linear Spline Models with Separate Slopes From −5 to −2 Years, −2 to 0, 0 to 2, and 2 to 5 Years Relative to Juridical Divorce.
**Figure S4**. Yearly Predicted and Observed Means of Psycholeptics, Antidepressants, GPs, Specialists, and Psychologist Visits by Conflict Group (*N* = 1784). Predicted Values are Based on Four‐Piece Linear Spline Models With Separate Slopes From −5 to −2 Years, −2 to 0, 0 to 2, and 2 to 5 Years Relative to Juridical Divorce.

## Data Availability

The information used in the analysis combines several Danish administrative registers and restricted data from an RCT (as described in the paper). The data use is subject to the European Union's General Data Protection Regulation (GDPR) per Danish regulations from May 2018. The data are physically stored on computers at Statistics Denmark and, due to security considerations, may not be transferred to computers outside Statistics Denmark. Researchers interested in obtaining access to the data employed in this paper are required to submit a written application to gain approval from Statistics Denmark. The application must include a detailed description of the proposed project, its purpose, and its social contribution as well as a description of the required datasets, variables and analysis population. Applications can be submitted by researchers who are affiliated with Danish institutions accepted by Statistics Denmark or by researchers outside of Denmark who collaborate with researchers affiliated with these institutions.
