# Supplementary material for: Divorce conflict and health across the divorce process: A 10‐year observational study of medicine prescriptions, primary care visits and hospitalisations
Source: Br J Health Psychol. 2026 Apr 24;31:e70075. doi: 10.1111/bjhp.70075 (PMC13107497; doi:10.1111/bjhp.70075)
Supplement: Supplementary file 1 — Table S1. Associations Between Self‐Rated Somatization, Depression, and Anxiety (SCL‐90‐R Subscales) and Medicine Prescriptions, Primary Care Visits, and Hospitalisations at the Year of Juridical Divorce (N = 1740). Table S2. Divorce conflict’s association with medicine prescriptions, primary care visits, and hospitalisations from divorce to 5 years after, controlling for outcomes from 5 to 3 years before divorce (N = 1784). Table S3. Divorce Conflict’s Association with Psycholeptics, Antidepressants, GP Visits, Specialist Visits, and Psychologist Visits from Divorce to 5 Years After, controlling for Outcomes from 5 to 3 Years Before Divorce (N = 1784). Table S4. Divorce conflict’s association with medicine prescriptions, primary care visits, and hospitalisations from divorce to 5 years after, controlling for outcomes from 5 to 2 years before divorce (N = 1784). Table S5. Divorce Conflict’s Association with Psycholeptics, Antidepressants, GP Visits, Specialist Visits, and Psychologist Visits from Divorce to 5 Years After, controlling for Outcomes from 5 to 2 Years Before Divorce (N = 1784). Table S6. Divorce conflict’s association with medicine prescriptions, primary care visits, and hospitalisations from 2 years before divorce to 5 years after, controlling for outcomes from 5 to 3 years before divorce (N = 1784). Table S7. Divorce Conflict’s Association with Psycholeptics, Antidepressants, GP Visits, Specialist Visits, and Psychologist Visits from 2 Years Before Divorce to 5 Years After, controlling for Outcomes from 5 to 3 Years Before Divorce (N = 1784). Table S8. Divorce Conflict’s Association with Psycholeptics, Antidepressants, GP Visits, Specialist Visits, and Psychologist Visits, Modelled Using Interactions Between Conflict Level and Two‐Piece Linear Splines for Time Before and After Divorce (N = 1784). Figure S1. Average Marginal Predicted Psycholeptics Prescriptions, Antidepressants Prescriptions, GP Visits, Specialist Visits, and Psychologist Visits by Co [file BJHP-31-0-s001.docx]

**Suppl. Table 1.** Associations Between Self-Rated Somatization, Depression, and Anxiety (SCL-90-R Subscales) and Medicine Prescriptions, Primary Care Visits, and Hospitalizations at the Year of Juridical Divorce (N=1,740)

| Self-rated measure (standardized) | **Medicine prescriptions IRR (CI 95%)** | **Primary care visits  IRR (CI 95%)** | **Hospitalizations  OR (CI 95%)** |
| --- | --- | --- | --- |
| Depression | 1.424** (1.205 to 1.683) | 1.157** (1.103 to 1.214) | 1.219 (0.984 to 1.510) |
| Anxiety | 1.572** (1.343 to 1.840) | 1.185** (1.132 to 1.240) | 1.119 (0.915 to 1.368) |
| Somatization | 1.479** (1.281 to 1.707) | 1.221** (1.169 to 1.276) | 1.255* (1.041 to 1.514) |

Notes: *p<0.05 ** p<0.01. All presented coefficients are estimated from the full models adjusted for divorce initiator, age, gender, income, and education. The somatization, depression, and anxiety subscales were standardized before analyses. Medicine prescriptions and primary care visits were analysed using negative binomial regression with exponentiated coefficients reported as Incidence Rate Ratios (IRRs); hospitalizations (binary outcome) were analysed using logistic regression with exponentiated coefficients reported as Odds Ratios (ORs).

**Suppl. Table 2.** Divorce conflict's association with medicine prescriptions, primary care visits, and hospitalizations from divorce to five years after, controlling for outcomes from five to three years before divorce (N=1,784).

| Health Outcome | IRR/OR | 95% CI | *P* value |
| --- | --- | --- | --- |
| **Medicine prescriptions (years 0 to 5)** | *IRR* |  |  |
| Conflict level | 1.275 | 1.106 to 1.469 | 0.001 |
| Medicine prescriptions (years -5 to -3) | 3.225 | 2.824 to 3.683 | <0.001 |
| Divorce initiator (ref: respondent) |  |  |  |
| Both | 1.178 | 0.711 to 1.952 | 0.524 |
| Former spouse | 1.352 | 0.980 to 1.863 | 0.066 |
| Age | 1.016 | 0.998 to 1.035 | 0.081 |
| Gender | 1.028 | 0.725 to 1.457 | 0.878 |
| Income | 0.999 | 0.999 to 0.999 | <0.001 |
| Education | 1.001 | 0.824 to 1.214 | 0.995 |
| **Primary care visits (years 0 to 5)** | *IRR* |  |  |
| Conflict level | 1.045 | 1.003 to 1.088 | 0.034 |
| Primary care visits (years -5 to -3) | 1.014 | 1.012 to 1.015 | <0.001 |
| Divorce initiator (ref: respondent) |  |  |  |
| Both | 1.014 | 0.916 to 1.124 | 0.784 |
| Former spouse | 0.942 | 0.875 to 1.014 | 0.113 |
| Age | 1.004 | 0.997 to 1.010 | 0.248 |
| Gender | 1.476 | 1.364 to 1.597 | <0.001 |
| Income | 0.999 | 0.999 to 1.000 | 0.015 |
| Education | 0.961 | 0.916 to 1.007 | 0.095 |
| **Ever hospitalized (years 0 to 5)** | *OR* |  |  |
| Conflict level | 1.131 | 1.009 to 1.269 | 0.035 |
| Ever hospitalized (years -5 to -3) | 2.436 | 1.813 to 3.273 | <0.001 |
| Divorce initiator (ref: respondent) |  |  |  |
| Both | 0.942 | 0.680 to 1.305 | 0.722 |
| Former spouse | 0.808 | 0.634 to 1.030 | 0.085 |
| Age | 1.009 | 0.996 to 1.023 | 0.186 |
| Gender | 1.167 | 0.914 to 1.489 | 0.215 |
| Income | 0.999 | 0.999 to 1.000 | 0.604 |
| Education | 0.850 | 0.731 to 0.987 | 0.033 |

Notes: All presented coefficients are estimated from the full models adjusted for divorce initiator, age, gender, income, and education. Medicine prescriptions and primary care visits were analysed using negative binomial regression, with exponentiated coefficients reported as incidence rate ratios (IRRs). Hospitalizations (binary outcome) were analysed using logistic regression, with exponentiated coefficients reported as odds ratios (ORs). Medicine prescriptions (years −5 to −3) were log-transformed.

**Suppl. Table 3.** Divorce Conflict's Association with Psycholeptics, Antidepressants, GP Visits, Specialist Visits, and Psychologist Visits from Divorce to Five Years After, Controlling for Outcomes from Five to Three Years Before Divorce (N=1,784)

| Health outcome | IRR | 95% CI | *P* value |
| --- | --- | --- | --- |
| **Psycholeptics (years 0 to 5)** |  |  |  |
| Conflict level | 1.572 | 1.308 to 1.889 | <0.001 |
| Psycholeptics (years -5 to -3) | 5.020 | 3.710 to 6.793 | <0.001 |
| **Antidepressants (years 0 to 5)** |  |  |  |
| Conflict level | 1.116 | 0.934 to 1.333 | 0.227 |
| Antidepressant (years -5 to -3) | 3.818 | 3.202 to 4.553 | <0.001 |
| **GP visits (years 0 to 5)** |  |  |  |
| Conflict level | 1.040 | 0.996 to 1.086 | 0.074 |
| GP visits (years -5 to -3) | 1.016 | 1.014 to 1.018 | <0.001 |
| **Specialist visits (years 0 to 5)** |  |  |  |
| Conflict level | 1.044 | 0.964 to 1.130 | 0.290 |
| Specialist visits (years -5 to -3) | 1.052 | 1.041 to 1.064 | <0.001 |
| **Psychologist visits (years 0 to 5)** |  |  |  |
| Conflict level | 1.237 | 1.068 to 1.434 | 0.005 |
| Psychologist visits (years -5 to -3) | 1.107 | 1.064 to 1.152 | <0.001 |

Notes: All presented coefficients are estimated from the full models adjusted for divorce initiator, age, gender, income, and education. All models are analysed with negative binomial regression, with exponentiated coefficients as Incidence Rate Ratios (IRRs). Psycholeptics (years -5 to -3) and Antidepressant (years -5 to -3) have been logarithmically transformed

**Suppl. Table 4.** Divorce conflict's association with medicine prescriptions, primary care visits, and hospitalizations from divorce to five years after, controlling for outcomes from five to three years before divorce (N=1,784).

| Health Outcome | IRR/OR | 95% CI | *P* value |
| --- | --- | --- | --- |
| **Medicine prescriptions (years 0 to 5)** | *IRR* |  |  |
| Conflict level | 1.193 | 1.037 to 1.373 | 0.014 |
| Medicine prescriptions (years -5 to -2) | 3.008 | 2.704 to 3.347 | <0.001 |
| Divorce initiator (ref: respondent) |  |  |  |
| Both | 1.073 | 0.673 to 1.709 | 0.767 |
| Former spouse | 1.415 | 1.016 to 1.971 | 0.040 |
| Age | 1.012 | 0.995 to 1.029 | 0.157 |
| Gender | 1.097 | 0.782 to 1.539 | 0.594 |
| Income | 0.999 | 0.998 to 0.999 | <0.001 |
| Education | 0.973 | 0.794 to 1.193 | 0.795 |
| **Primary care visits (years 0 to 5)** | *IRR* |  |  |
| Conflict level | 1.035 | 0.996 to 1.075 | 0.075 |
| Primary care visits (years -5 to -2) | 1.012 | 1.011 to 1.013 | <0.001 |
| Divorce initiator (ref: respondent) |  |  |  |
| Both | 1.017 | 0.920 to 1.122 | 0.750 |
| Former spouse | 0.961 | 0.897 to 1.029 | 0.252 |
| Age | 1.002 | 0.996 to 1.008 | 0.553 |
| Gender | 1.438 | 1.333 to 1.550 | <0.001 |
| Income | 0.999 | 0.999 to 1.000 | 0.019 |
| Education | 0.963 | 0.921 to 1.006 | 0.092 |
| **Ever hospitalized (years 0 to 5)** | *OR* |  |  |
| Conflict level | 1.126 | 1.003 to 1.265 | 0.045 |
| Ever hospitalized (years -5 to -2) | 2.681 | 2.066 to 3.478 | <0.001 |
| Divorce initiator (ref: respondent) |  |  |  |
| Both | 0.950 | 0.684 to 1.319 | 0.759 |
| Former spouse | 0.804 | 0.630 to 1.026 | 0.080 |
| Age | 1.010 | 0.997 to 1.024 | 0.135 |
| Gender | 1.135 | 0.887 to 1.451 | 0.314 |
| Income | 1.000 | 0.999 to 1.000 | 0.586 |
| Education | 0.867 | 0.741 to 1.001 | 0.051 |

Notes: All presented coefficients are estimated from the full models adjusted for divorce initiator, age, gender, income, and education. Medicine prescriptions and primary care visits were analysed using negative binomial regression, with exponentiated coefficients reported as incidence rate ratios (IRRs). Hospitalizations (binary outcome) were analysed using logistic regression, with exponentiated coefficients reported as odds ratios (ORs). Medicine prescriptions (years −5 to −2) were log-transformed.

**Suppl. Table 5.**  Divorce Conflict's Association with Psycholeptics, Antidepressants, GP Visits, Specialist Visits, and Psychologist Visits from Divorce to Five Years After, Controlling for Outcomes from Five to Two Years Before Divorce (N=1,784)

| Health outcome | IRR | 95% CI | *P* value |
| --- | --- | --- | --- |
| **Psycholeptics (years 0 to 5)** |  |  |  |
| Conflict level | 1.450 | 1.194 to 1.762 | <0.001 |
| Psycholeptics (years -5 to -2) | 4.157 | 3.381 to 5.110 | <0.001 |
| **Antidepressants (years 0 to 5)** |  |  |  |
| Conflict level | 1.056 | 0.869 to 1.284 | 0.584 |
| Antidepressant (years -5 to -2) | 3.407 | 2.962 to 3.919 | <0.001 |
| **GP visits (years 0 to 5)** |  |  |  |
| Conflict level | 1.032 | 0.991 to 1.074 | 0.127 |
| GP visits (years -5 to -2) | 1.014 | 1.012 to 1.015 | <0.001 |
| **Specialist visits (years 0 to 5)** |  |  |  |
| Conflict level | 1.039 | 0.961 to 1.124 | 0.333 |
| Specialist visits (years -5 to -2) | 1.046 | 1.038 to 1.054 | <0.001 |
| **Psychologist visits (years 0 to 5)** |  |  |  |
| Conflict level | 1.230 | 1.064 to 1.422 | 0.005 |
| Psychologist visits (years -5 to -2) | 1.119 | 1.078 to 1.161 | <0.001 |

Notes: All presented coefficients are estimated from the full models adjusted for divorce initiator, age, gender, income, and education. All models are analysed with negative binomial regression, with exponentiated coefficients as Incidence Rate Ratios (IRRs). Psycholeptics (years -5 to -2) and Antidepressant (years -5 to -2) have been logarithmically transformed.

**Suppl. Table 6.** Divorce conflict's association with medicine prescriptions, primary care visits, and hospitalizations from divorce to five years after, controlling for outcomes from five to three years before divorce (N=1,784).

| Health Outcome | IRR/OR | 95% CI | *P* value |
| --- | --- | --- | --- |
| **Medicine prescriptions (years -2 to 5)** | *IRR* |  |  |
| Conflict level | 1.271 | 1.105 to 1.462 | 0.001 |
| Medicine prescriptions (years -5 to -3) | 3.503 | 3.086 to 3.976 | <0.001 |
| Divorce initiator (ref: respondent) |  |  |  |
| Both | 1.291 | 0.793 to 2.100 | 0.305 |
| Former spouse | 1.348 | 0.988 to 1.838 | 0.060 |
| Age | 1.017 | 0.999 to 1.035 | 0.072 |
| Gender | 1.024 | 0.733 to 1.433 | 0.888 |
| Income | 0.999 | 0.999 to 1.000 | <0.001 |
| Education | 0.996 | 0.822 to 1.193 | 0.920 |
| **Primary care visits (years -2 to 5)** | *IRR* |  |  |
| Conflict level | 1.039 | 1.003 to 1.076 | 0.033 |
| Primary care visits (years -5 to -3) | 1.014 | 1.013 to 1.016 | <0.001 |
| Divorce initiator (ref: respondent) |  |  |  |
| Both | 0.999 | 0.912 to 1.093 | 0.979 |
| Former spouse | 0.942 | 0.882 to 1.007 | 0.079 |
| Age | 1.004 | 0.999 to 1.010 | 0.140 |
| Gender | 1.417 | 1.321 to 1.520 | <0.001 |
| Income | 1.000 | 0.999 to 1.000 | 0.011 |
| Education | 0.967 | 0.928 to 1.009 | 0.114 |
| **Ever hospitalized (years -2 to 5)** | *OR* |  |  |
| Conflict level | 1,173 | 1.055 to 1.304 | 0.003 |
| Ever hospitalized (years -5 to -3) | 2.239 | 1.672 to 2.997 | <0.001 |
| Divorce initiator (ref: respondent) |  |  |  |
| Both | 0.970 | 0.717 to 1.314 | 0.845 |
| Former spouse | 0.789 | 0.629 to 0.996 | 0.041 |
| Age | 0.997 | 0.984 to 1.010 | 0.619 |
| Gender | 1.290 | 1.028 to 1.619 | 0.028 |
| Income | 1.000 | 0.999 to 1.000 | 0.633 |
| Education | 0.794 | 0.690 to 0.912 | 0.001 |

Notes: All presented coefficients are estimated from the full models adjusted for divorce initiator, age, gender, income, and education. Medicine prescriptions and primary care visits were analysed using negative binomial regression, with exponentiated coefficients reported as incidence rate ratios (IRRs). Hospitalizations (binary outcome) were analysed using logistic regression, with exponentiated coefficients reported as odds ratios (ORs). Medicine prescriptions (years −5 to −3) were log-transformed.

**Suppl. Table 7.** Divorce Conflict's Association with Psycholeptics, Antidepressants, GP Visits, Specialist Visits, and Psychologist Visits from Two Years Before Divorce to Five Years After, Controlling for Outcomes from Five to Three Years Before Divorce (N=1,784)

| Health outcome | IRR | 95% CI | *P* value |
| --- | --- | --- | --- |
| **Psycholeptics (years -2 to 5)** |  |  |  |
| Conflict level | 1.538 | 1.303 to 1.817 | <0.001 |
| Psycholeptics (years -5 to -3) | 5.501 | 4.128 to 7.330 | <0.001 |
| **Antidepressants (years -2 to 5)** |  |  |  |
| Conflict level | 1.148 | 0.963 to 1.369 | 0.124 |
| Antidepressant (years -5 to -3) | 4.257 | 3.587 to 5.053 | <0.001 |
| **GP visits (years -2 to 5)** |  |  |  |
| Conflict level | 1.032 | 0.994 to 1.071 | 0.099 |
| GP visits (years -5 to -3) | 1.016 | 1.015 to 1.018 | <0.001 |
| **Specialist visits (years -2 to 5)** |  |  |  |
| Conflict level | 1.043 | 0.974 to 1.117 | 0.232 |
| Specialist visits (years -5 to -3) | 1.061 | 1.050 to 1.072 | <0.001 |
| **Psychologist visits (years -2 to 5)** |  |  |  |
| Conflict level | 1.211 | 1.053 to 1.394 | 0.007 |
| Psychologist visits (years -5 to -3) | 1.141 | 1.097 to 1.187 | <0.001 |

Notes: All presented coefficients are estimated from the full models adjusted for divorce initiator, age, gender, income, and education. All models are analysed with negative binomial regression, with exponentiated coefficients reported as Incidence Rate Ratios (IRRs). Psycholeptics (years -5 to -3) and Antidepressant (years -5 to -3) have been logarithmically transformed.

**Suppl. Table 8.** Divorce Conflict’s Association with Psycholeptics, Antidepressants, GP Visits, Specialist Visits, and Psychologist Visits, Modelled Using Interactions Between Conflict Level and Two-Piece Linear Splines for Time Before and After Divorce (N = 1,784)

|  | **Psycholeptics IRR (CI 95%)** | **Antidepressants  IRR (CI 95%)** | **GP visits IRR (CI 95%)** | **Specialist visits  IRR (CI 95%)** | **Psychologist visits IRR (CI 95%)** |
| --- | --- | --- | --- | --- | --- |
| **Conflict level main effect** |  |  |  |  |  |
| Average conflict vs. low conflict | **1.895 (1.221**–**2.941)** | 1.315 (0.895–1.931) | 0.961 (0.879–1.049) | 0.976 (0.782–1.220) | 1.037 (0.581–1.851) |
| High conflict vs. low conflict | **2.569 (1.482**–**4.454)** | 1.574 (0.969–2.556) | **1.227 (1.097–1.373)** | 1.197 (0.898–1.595) | 1.834 (0.896–3.752) |
| **Pre-divorce slope for low conflict** | **1.413 (1.183–1.689)** | 1.133 (0.983–1.306) | 0.999 (0.969–1.030) | 1.027 (0.951–1.110) | **1.257 (1.027–1.538)** |
| **Post-divorce slope for low conflict** | 0.904 (0.787–1.038) | 1.001 (0.888–1.276) | **0.890 (0.865–0.916)** | **0.930 (0.865–0.999)** | **0.689 (0.569–0.835)** |
| **Conflict level x pre-divorce slope** |  |  |  |  |  |
| Average conflict vs. low conflict | 0.971 (0.799–1.179) | 0.994 (0.850–1.163) | 1.003 (0.969–1.038) | 1.012 (0.929–1.103) | 1.038 (0.832–1.295) |
| High conflict vs. low conflict | 0.976 (0.767–1.243) | 0.924 (0.759–1.124) | 1.040 (0.995–1.086) | 1.047 (0.937–1.170) | 1.291 (0.972–1.715) |
| **Conflict level x post-divorce slope** |  |  |  |  |  |
| Average conflict vs. low conflict | 1.049 (0.901–1.222) | 0.943 (0.826–1.076) | 1.008 (0.976–1.041) | 0.962 (0.888–1.043) | 1.049 (0.850–1.294) |
| High conflict vs. low conflict | 1.121 (0.928–1.355) | 0.922 (0.780–1.090) | 0.975 (0.937–1.016) | 0.945 (0.851–1.049) | 0.956 (0.737–1.241) |

Notes: p<0.05 highlighted in bold. “x” denotes an interaction term that reflects whether the slope for each time segment differs by conflict level compared to the low conflict group. All presented coefficients are estimates from the full models adjusted for divorce initiator, age, gender, income, and education. All models are analysed with negative binomial regression, with exponentiated coefficients reported as Incidence Rate Ratios. All models include interactions between conflict level (low, average, high) and two-piece linear spline time variables (pre-divorce and post-divorce slopes).


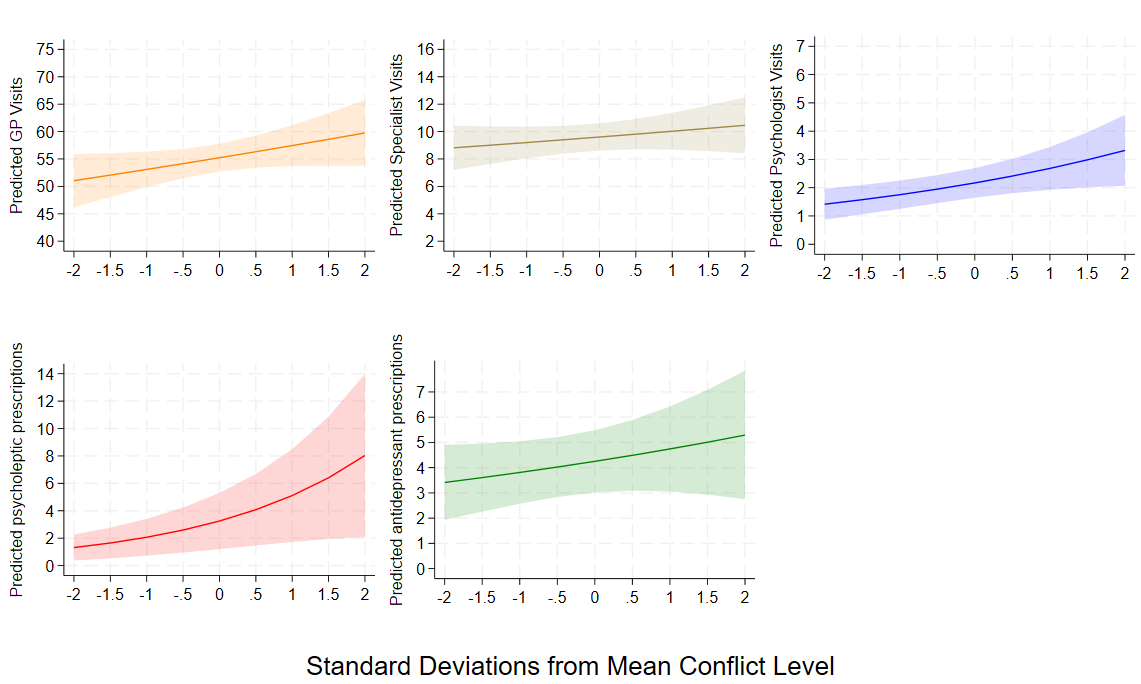


**Suppl. Figure 1.** Average Marginal Predicted Psycholeptics Prescriptions, Antidepressants Prescriptions, GP Visits, Specialist Visits, and Psychologist Visits by Conflict Level, with 95% Confidence Intervals (N=1,784)


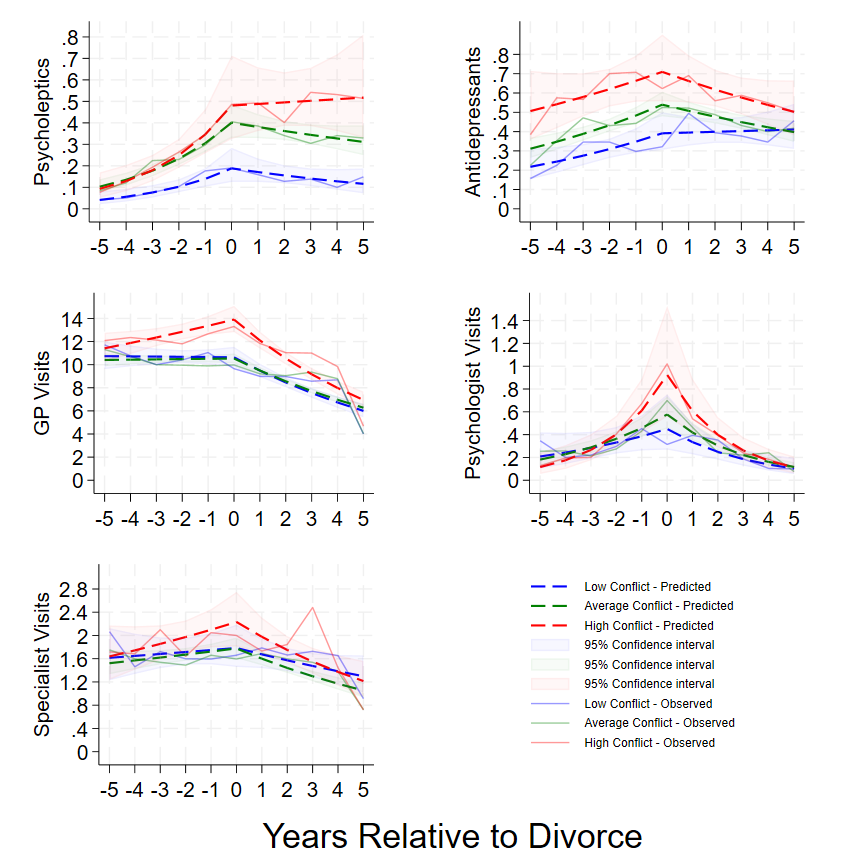


**Suppl. Figure 2.** Yearly Predicted and Observed Means of Psycholeptics, Antidepressants, GPs, Specialists, and Psychologist Visits by Conflict Group (N=1,784). Predicted Values Are Based on Two-Piece Linear Models With Separate Slopes Before and After Juridical Divorce (Year 0).

**Suppl.
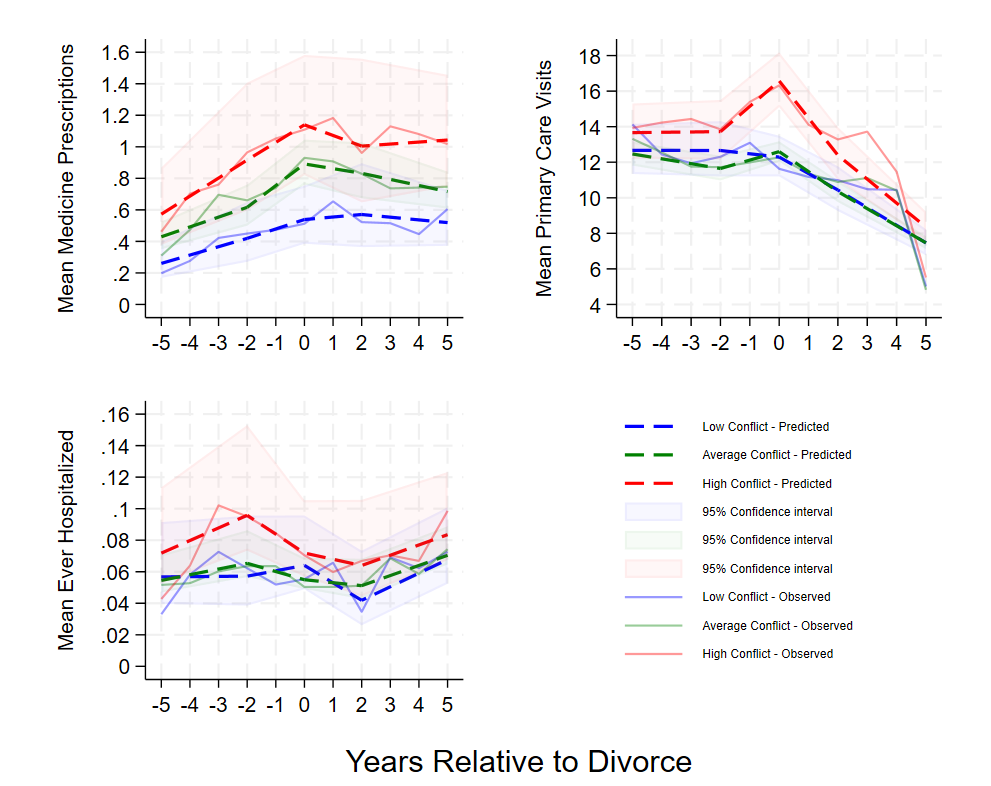
Figure 3**. Yearly Predicted and Observed Means of Medicine Prescriptions, Primary Care Visits, and Binary Hospitalizations by Conflict Group (N=1,784). Predicted Values are Based on Four-Piece Linear Spline Models with Separate Slopes From –5 to –2 Years, –2 to 0, 0 to 2, and 2 to 5 Years Relative to Juridical Divorce.

**
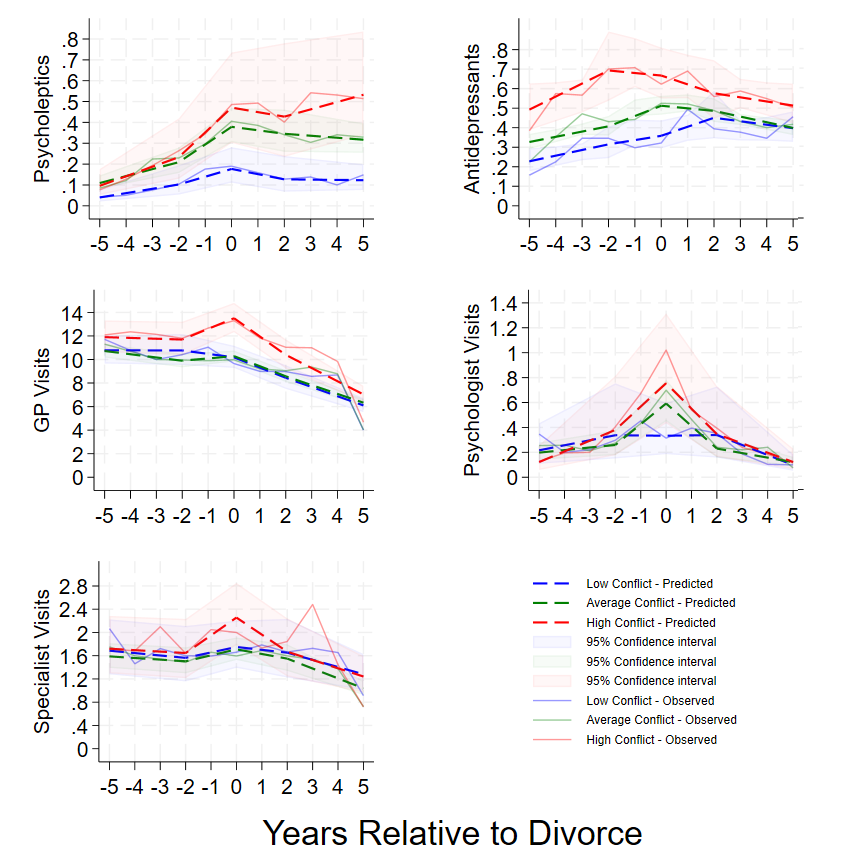
**

**Suppl. Figure 4.** Yearly Predicted and Observed Means of Psycholeptics, Antidepressants, GPs, Specialists, and Psychologist Visits by Conflict Group (N=1,784). Predicted Values are Based on Four-Piece Linear Spline Models With Separate Slopes From –5 to –2 Years, –2 to 0, 0 to 2, and 2 to 5 Years Relative to Juridical Divorce.
